# Candidate Biomarkers Linking Periodontitis with Atherosclerotic and Related Cardiovascular Phenotypes: A Systematic Review Focused on Sex-Specific Evidence

**DOI:** 10.3390/ijms27146495

**Published:** 2026-07-22

**Authors:** Marco Severino, Angelo Michele Inchingolo, Francesca Calò, Claudia Ciocia, Francesco Inchingolo, Grazia Marinelli, Arianna Viarchi, Claudia Theodora Truppa, Andrea Palermo, Ioana Roxana Bordea, Alessio Danilo Inchingolo, Gianna Dipalma

**Affiliations:** 1 Department of Medicine and Surgery, University of Perugia, 06132 Perugia, Italy; marco.severino@unipg.it (M.S.); angelo.inchingolo@unipg.it (A.M.I.); 2 Interdisciplinary Department of Medicine, University of Bari “Aldo Moro”, 70124 Bari, Italy; francesca.calo@uniba.it (F.C.); claudia.ciocia@uniba.it (C.C.); graziamarinelli@live.it (G.M.); alessiodanilo.inchingolo@uniba.it (A.D.I.); giannadipalma@tiscali.it (G.D.); 3 Department of Life, Health and Environmental Sciences, University of L’Aquila, 67100 L’Aquila, Italy; arianna.viarchi@graduate.univaq.it (A.V.); claudiatheodora.truppa@graduate.univaq.it (C.T.T.); 4 Department of Experimental Medicine, University of Salento, 73100 Lecce, Italy; andrea.palermo@unisalento.it; 5 Department of Oral Rehabilitation, Faculty of Dentistry, Iuliu Hațieganu University of Medicine and Pharmacy, 400012 Cluj-Napoca, Romania

**Keywords:** atherosclerosis, periodontitis, cardiovascular disease, blood biomarkers, inflammation, endothelial dysfunction, menopause, candidate biomarkers, sex-related evidence

## Abstract

Periodontitis and cardiovascular disease share inflammatory, immune, endothelial, oxidative, and lipid-related pathways. Although sex-related differences are well established for many cardiovascular biomarkers, it remains unclear whether sex modifies biomarker patterns at the intersection of periodontal and cardiovascular disease. PubMed, Scopus, and Web of Science were searched in accordance with PRISMA 2020. Eligible adult human studies evaluated periodontitis or periodontal inflammation together with atherosclerotic or related cardiovascular phenotypes and reported candidate biomarkers in blood or, as supportive evidence, oral fluids. High-throughput omics studies were eligible when available. Data on study design, periodontal and cardiovascular assessment, biological matrix, biomarker findings, sex-stratified analyses, and menopausal status were extracted. Twelve studies were included. Most assessed single biomarkers or small predefined panels rather than discovery-scale omics. Reported markers involved inflammation and immunity (CRP, hs-CRP, IL-6, LPS, and periodontal antibodies), endothelial or vascular injury, lipid and autoimmune pathways (PCSK9 and anti-ApoA-1 IgG), innate-immune and metabolic regulation (MBL and SIRT1), cardiac stress, and oxidative stress. Findings were heterogeneous across cardiovascular phenotypes, and several key associations were null or imprecise. Only two studies performed direct male–female comparisons: one enrolled women only, and none stratified women by menopausal status. No study derived and validated a sex-specific omics signature. The literature identifies overlapping candidate-biomarker pathways but does not establish a causal, clinically validated, sex-specific, or menopause-specific signature of periodontitis-associated cardiovascular disease. The current evidence should be considered hypothesis-generating.

## 1. Introduction

Cardiovascular diseases remain the leading cause of mortality worldwide and are responsible for a substantial global clinical and economic burden. According to the World Health Organization, cardiovascular diseases caused an estimated 19.8 million deaths in 2022, accounting for approximately 32% of all global deaths, with most deaths attributable to myocardial infarction and stroke. Atherosclerosis represents the major pathological substrate underlying many cardiovascular events and is now recognized as a chronic inflammatory disease involving endothelial dysfunction, lipid accumulation, immune-cell activation, oxidative stress and vascular remodeling [1,2,3,4,5].

Periodontitis is a highly prevalent chronic inflammatory disease characterized by a dysbiotic microbial biofilm, destruction of periodontal tissues and systemic inflammatory consequences. In recent years, increasing attention has been directed toward the relationship between periodontitis and cardiovascular disease. The consensus report of the European Federation of Periodontology and the World Heart Federation highlighted a significant body of evidence supporting an independent association between severe periodontitis and cardiovascular diseases, particularly atherosclerotic cardiovascular disease [6,7,8,9,10].

Several biological mechanisms may explain the link between periodontal inflammation and atherosclerosis. Periodontal pathogens and their virulence factors may enter the bloodstream, promoting systemic inflammation, endothelial activation and immune dysregulation. In parallel, increased circulating levels of inflammatory mediators, oxidative stress markers and matrix remodeling enzymes may contribute to vascular injury and plaque instability. Candidate biomarkers involved in this oral-vascular inflammatory axis include C-reactive protein, interleukin-6, tumor necrosis factor-alpha, myeloperoxidase, matrix metalloproteinases and lipid-related mediators [11,12,13,14].

Despite the biological plausibility of the oral–vascular link, the molecular evidence remains fragmented. A distinction is required between candidate-biomarker studies and high-throughput omics. Candidate-biomarker studies quantify one or a limited number of predefined analytes using targeted immunoassays or related laboratory methods. By contrast, high-throughput proteomics, transcriptomics, metabolomics, and integrated multi-omics are discovery-oriented approaches that profile broad molecular systems. Most studies available at the intersection of periodontitis and cardiovascular disease belong to the first category rather than the second [15,16,17,18].

Sex is a biologically relevant variable in cardiovascular research. In the Framingham Heart Study, 61 of 71 circulating cardiovascular biomarkers differed between men and women, and several differences were attenuated after menopause [19,20,21,22,23]. However, evidence of sex-related differences in general cardiovascular biomarker concentrations does not establish that sex modifies the association between periodontitis and cardiovascular disease [24,25,26,27]. A meta-analysis of periodontal disease and cardiovascular outcomes found significant associations in both men and women but no statistically significant difference between the sexes; the certainty of this evidence was very low [28,29,30,31,32,33,34,35,36].

These observations address different levels of inference and are therefore not contradictory. Baseline biomarker concentrations may differ by sex because of hormonal, metabolic, body-composition, or vascular factors, while the additional contribution of periodontal inflammation may be similar in men and women. A periodontitis-specific sex effect can only be demonstrated through adequately powered interaction analyses or sex-stratified longitudinal studies that use harmonized periodontal definitions, standardized cardiovascular phenotyping, and comparable biomarker assays [37,38,39,40,41].

Menopausal status may further modify inflammatory, lipid, endothelial, and immune pathways. Nevertheless, menopause cannot be inferred from age alone and should be recorded directly. The available studies rarely reported reproductive variables and did not provide a consistent comparison among men, premenopausal women, and postmenopausal women. Consequently, menopause-related biomarker differences remain a research gap rather than an evidence-supported finding [42,43,44,45,46,47].

Accordingly, this systematic review aimed to map candidate biomarkers reported at the intersection of periodontitis and atherosclerotic or related cardiovascular phenotypes and to determine whether the available evidence supports sex- or menopause-specific biomarker differences [48,49,50,51,52,53]. High-throughput omics evidence was sought when available, but it was not assumed to be present.

## 2. Methods

This systematic review was conducted according to the Preferred Reporting Items for Systematic Reviews and Meta-Analyses (PRISMA) guidelines and was registered in the International Prospective Register of Systematic Reviews (PROSPERO) under registration number CRD420261420329.

### 2.1. Eligibility Criteria

The PECOS framework was used to define the eligibility criteria based on the following research question:

“In adults with periodontitis or periodontal inflammation and atherosclerotic or related cardiovascular phenotypes, which circulating or oral-fluid candidate biomarkers have been evaluated, and to what extent does the available evidence support differences by sex or menopausal status?”

The PECOS model was structured as follows:

- (P) Population: Adults with established atherosclerotic cardiovascular disease, subclinical atherosclerosis, or related cardiovascular or vascular phenotypes.
- (E) Exposure: Periodontitis, periodontal inflammation, periodontal pathogens, or periodontal treatment, as defined by the individual study.
- (C) Comparison: Participants without periodontitis, with a lower periodontal inflammatory burden, receiving a control periodontal intervention, or belonging to a comparator cardiovascular group; sex-based comparisons were extracted when available.
- (O) Outcomes: Candidate biomarkers measured in blood, serum, plasma, peripheral blood cells, saliva, or gingival crevicular fluid; vascular imaging or functional outcomes when directly linked to biomarker assessment; sex-stratified findings and menopausal status as secondary outcomes. Genuine high-throughput proteomic, transcriptomic, metabolomic, or multi-omics data were eligible when available.
- (S) Study design: Human observational studies and randomized or non-randomized clinical studies with sufficient methodological detail. Reviews, purely in vitro studies, animal studies, and unvalidated bioinformatic hypothesis-generating analyses were excluded from the primary synthesis.

### 2.2. Inclusion Criteria

This review included observational cross-sectional, retrospective, prospective, cohort, case–control, clinical, omics-based and validated bioinformatic studies involving adult human subjects. Eligible studies investigated the association between periodontitis or periodontal inflammation and atherosclerosis, cardiovascular disease, atherosclerotic plaque or atherosclerotic risk phenotypes. No restrictions were imposed with regard to sex, ethnicity or geographical area. The availability of sex-specific data was not required for study inclusion. Sex distribution, sex-stratified analyses, single-sex populations, and menopausal status were recorded when reported.

The reviewers evaluated all relevant papers according to the following inclusion criteria:

1. Human subject studies involving adults;
2. Full-text articles published in English;
3. Studies evaluating periodontitis, periodontal inflammation or periodontal pathogens in association with established atherosclerotic cardiovascular disease, subclinical atherosclerosis, vascular imaging markers, endothelial dysfunction or vascular risk phenotypes potentially related to atherosclerotic burden;
4. Studies reporting at least one eligible circulating or oral-fluid candidate biomarker, or a high-throughput omics profile, in relation to periodontal and cardiovascular phenotypes.
5. Studies analyzing blood, plasma, serum, peripheral blood cells, saliva, gingival crevicular fluid, or vascular tissue when the biological matrix was directly relevant to the periodontal–cardiovascular question.
6. Sex distribution, sex-stratified results, and menopausal status were extracted when available; their absence did not constitute an exclusion criterion because the sufficiency of sex-specific evidence was itself an outcome of the review.
7. Studies published between 1 January 2013 and 1 May 2026.

### 2.3. Exclusion Criteria

Studies were excluded if they met any of the following criteria:

1. Articles written in languages other than English;
2. Studies not involving human subjects;
3. Studies conducted exclusively in animals or in vitro;
4. Studies involving paediatric or adolescent populations;
5. Off-topic studies not addressing periodontitis, periodontal inflammation, atherosclerosis, cardiovascular disease or related atherosclerotic risk phenotypes;
6. Reviews, narrative reviews, letters to the editor, comments, editorials, expert opinions and conference abstracts without sufficient methodological details. Systematic reviews and meta-analyses were not included in the main evidence synthesis but were used to support the background, contextual interpretation and manual screening of reference lists.
7. Studies with ineligible outcome measures, ineligible populations or insufficient information regarding study design, biological samples, biomarker assessment or outcome definition;
8. Retracted studies or studies with formal expressions of concern.

### 2.4. Search Strategy

A systematic literature search was conducted in PubMed, Scopus and Web of Science to identify relevant English-language studies published between 1 January 2013 and 1 May 2026. The search was designed to retrieve articles investigating the relationship between periodontal disease, atherosclerotic or related cardiovascular phenotypes and candidate biomarkers or high-throughput omics approaches.

The search strategy combined three main concept blocks: periodontal disease, atherosclerotic cardiovascular disease or related vascular phenotypes and candidate biomarkers or high-throughput omics approaches. The following core Boolean search string was used and adapted to the syntax of each database:

(periodontitis OR “periodontal disease” OR “periodontal inflammation” OR “periodontal infection” OR “periodontal pathogens” OR “*Porphyromonas gingivalis*” OR “*Aggregatibacter actinomycetemcomitans*” OR “clinical attachment loss” OR “bleeding on probing”) AND (atherosclerosis OR “atherosclerotic plaque” OR “cardiovascular disease” OR “coronary artery disease” OR “myocardial infarction” OR stroke OR “ischemic stroke” OR “carotid intima-media thickness” OR “carotid plaque” OR “endothelial dysfunction” OR hypertension OR prehypertension OR “vascular inflammation”) AND (biomarker OR biomarkers OR “blood biomarker” OR “serum biomarker” OR “plasma biomarker” OR “inflammatory marker” OR “immune marker” OR “endothelial marker” OR omics OR proteomic OR proteomics OR proteome OR immunoproteomic OR immunoproteomics OR transcriptomic OR transcriptomics OR transcriptome OR “gene expression” OR “RNA sequencing” OR “multi-omics” OR “targeted proteomics”).

Terms related to sex, gender and menopausal status were not included in the primary search string because sex-specific evidence was evaluated as a secondary objective and its absence was considered an informative finding. During full-text screening and data extraction, the reviewers recorded whether each study reported sex distribution, direct sex-stratified estimates, formal interaction analyses, single-sex data, and menopausal status. After duplicate removal, titles and abstracts were independently screened by the reviewers. Potentially eligible articles were then retrieved in full text and assessed according to the predefined inclusion and exclusion criteria. In addition, the reference lists of included studies and relevant systematic reviews were manually screened to identify further eligible articles.

The complete database-specific search strategies, including the search fields used for each database, are reported in Table 1.

### 2.5. Study Selection

The study selection process was carried out in two phases. In the first phase, two reviewers (F.I. and A.P.) independently screened the titles and abstracts of all retrieved records to identify potentially eligible studies. Articles that clearly did not meet the inclusion criteria were excluded. In the second phase, the same reviewers (F.I. and A.P.) independently assessed the full texts of the preselected articles to determine final eligibility.

Any disagreements between the two reviewers were resolved through discussion. If consensus could not be reached, a third reviewer (A.M.I.) was consulted to make the final decision. Before the screening process, the reviewers were calibrated to ensure consistency and improve inter-reviewer agreement.

### 2.6. Data Extraction

Two reviewers (F.I. and A.M.I.) independently extracted data from the included studies. Any discrepancies were resolved through discussion; when consensus could not be reached, a third reviewer (A.P.) was consulted.

The following information was extracted using a standardized form: study design and setting; sample characteristics; periodontal definition and assessment method; cardiovascular phenotype and assessment; biological matrix; assay or analytical platform; individual biomarkers and direction of findings; adjusted and unadjusted estimates; prespecified confounders; null results; sex distribution; direct sex-stratified estimates; formal sex-by-periodontitis interaction analyses; menopausal and reproductive information; and study limitations. Candidate-biomarker studies were distinguished from genuine high-throughput omics studies according to the breadth and discovery-oriented nature of the analytical platform. When essential data were missing or insufficient to assess eligibility or outcomes, the study was excluded from the final qualitative synthesis.

### 2.7. Quality Assessment

Two reviewers (F.I. and A.M.I.) independently assessed the risk of bias for the result from each included study that was most directly relevant to the review question. Before the assessment, the relevant exposure–outcome result was identified for each study to ensure that the judgments referred to a specific estimate rather than generically to the entire publication.

Observational studies, including cohort, cross-sectional, case–control, post hoc, and subgroup analyses, were evaluated using the Risk Of Bias In Non-randomized Studies of Exposures (ROBINS-E) tool. The assessment considered seven domains: bias due to confounding; bias arising from measurement of the exposure; bias in selection of participants into the study or analysis; bias due to post-exposure interventions; bias due to missing data; bias arising from measurement of the outcome; and bias in selection of the reported result. For cross-sectional, case–control, post hoc, and subgroup analyses, particular attention was given to temporality, participant selection, control of shared periodontal and cardiovascular confounders, and the possibility of selective reporting.

Randomized clinical trials were assessed using the Cochrane Risk of Bias 2 tool (RoB 2). The assessment considered five domains: bias arising from the randomization process; bias due to deviations from intended interventions; bias due to missing outcome data; bias in measurement of the outcome; and bias in selection of the reported result.

Domain-level and overall judgments were recorded separately according to study design and are summarized in Table 2, with Panel A reporting the ROBINS-E assessment and Panel B reporting the RoB 2 assessment. Any disagreements between the two reviewers were resolved through discussion; when consensus could not be reached, a third reviewer (A.P.) was consulted.

Risk-of-bias findings were incorporated into the qualitative synthesis. Findings from studies with greater concerns regarding confounding, participant selection, missing data, or selective reporting were interpreted more cautiously and were considered exploratory rather than confirmatory. Limited sample size was not treated as a risk-of-bias domain in itself but was considered a source of imprecision and reduced certainty when interpreting the overall evidence.

## 3. Results

### 3.1. Study Selection and Characteristics

**Figure 1:**
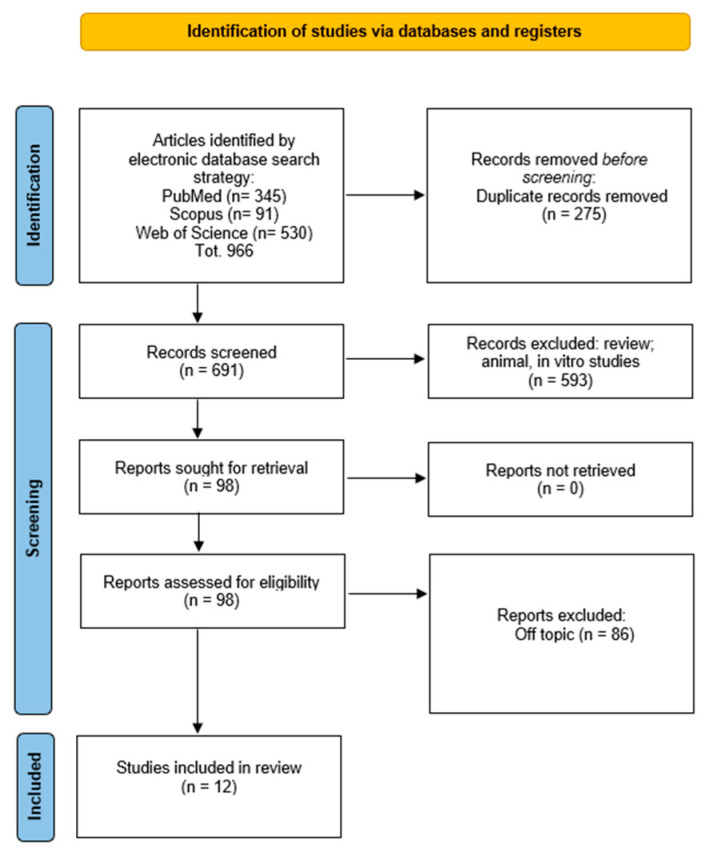
PRISMA flow diagram of the study selection process.

Figure 1 shows the PRISMA flow diagram of the study selection process. The diagram reports the number of records identified, screened, excluded and finally included in the qualitative synthesis according to the Preferred Reporting Items for Systematic Reviews and Meta-Analyses (PRISMA) guidelines. A total of 966 records were identified through database searching: PubMed (n = 345), Web of Science (n = 530) and Scopus (n = 91). After removal of 275 duplicate records, 691 records remained for title and abstract screening. Of these, 593 records were excluded because they were reviews, animal studies, in vitro studies or clearly outside the scope of the review. A total of 98 full-text reports were assessed for eligibility. Eighty-six reports were excluded because they did not meet the predefined inclusion criteria, mainly due to off-topic populations, outcomes or biomarker assessments. Finally, 12 studies were included in the qualitative synthesis. The study selection process is summarized in Figure 1.

The main characteristics, cardiovascular phenotypes, biomarker assessments and sex-specific information of the included studies are summarized in Table 3. Because the eligible studies included heterogeneous cardiovascular phenotypes, they were grouped according to the main cardiovascular or vascular condition investigated: established atherosclerotic cardiovascular disease, including coronary artery disease, myocardial infarction and ischemic stroke; subclinical atherosclerosis or vascular imaging markers, including carotid intima-media thickness and carotid plaque; vascular risk phenotypes, including hypertension and prehypertension; other vascular inflammatory conditions potentially related to atherosclerotic burden, including abdominal aortic aneurysm. This classification was used to support the qualitative interpretation of the evidence and to avoid pooling biologically and clinically heterogeneous outcomes.

Most included studies assessed one or a limited number of predefined candidate biomarkers rather than broad high-throughput omics profiles. Only two studies, Latorre Uriza et al. and Stănescu et al., reported direct comparisons between men and women. Da Venezia et al. enrolled women only and therefore did not permit a between-sex comparison. The remaining studies reported sex as a demographic characteristic or adjustment variable without providing sex-stratified biomarker estimates. No included study analyzed menopausal status. Accordingly, the evidence was synthesized as a map of overlapping candidate biomarkers and as an assessment of the absence, rather than the presence, of a validated sex-specific signature.

### 3.2. Risk of Bias

The risk of bias across the included studies was evaluated using design-appropriate tools and is summarized in Table 2, which is organized into two panels according to study design: observational studies assessed using ROBINS-E and randomized trials assessed using RoB 2.

Among the observational studies, the most frequent methodological concerns involved residual confounding and the selection of participants into the study or analysis. These concerns were particularly relevant in cross-sectional, case–control, post hoc, and pilot investigations, in which adjustment for age, smoking, adiposity, medication use, socioeconomic factors, periodontal severity, and baseline cardiovascular risk was incomplete or inconsistent. Exposure and outcome measurements were generally based on clinical periodontal examinations, laboratory assays, or vascular imaging and were therefore judged more favorably. Nevertheless, heterogeneous periodontal definitions and non-standardized biomarker assays reduced comparability across studies. Concerns regarding selection of the reported result were also considered when multiple biomarkers, subgroups, or assessment time points were analyzed without a clearly prespecified analytical plan.

The randomized trials were judged as having some concerns overall. Objective laboratory and vascular outcomes reduced concerns regarding outcome measurement; however, incomplete reporting of allocation procedures, possible deviations from the intended periodontal interventions, missing outcome data, multiple endpoints, and selective emphasis on within-group rather than between-group changes reduced confidence in the findings. These methodological limitations were considered when interpreting the reported effects of periodontal treatment on vascular and biomarker outcomes.

The risk-of-bias judgments were incorporated into the qualitative synthesis rather than being considered separately from the scientific interpretation. Findings from studies with greater concerns regarding confounding, participant selection, or selective reporting were interpreted as exploratory and hypothesis-generating. No included study provided sufficiently robust evidence to establish a causal association or a clinically validated sex-specific or menopause-specific biomarker signature. Limited sample size was not considered a risk-of-bias domain in itself but was taken into account as a source of imprecision and reduced certainty of the evidence.

### 3.3. Summary of Results

The included studies evaluated a heterogeneous set of candidate biomarkers across established cardiovascular events, subclinical vascular measures, treatment-related vascular outcomes, and broader cardiovascular phenotypes. Most studies measured one analyte or a small predefined panel; none used a discovery-and-validation workflow capable of establishing a comprehensive sex-specific proteomic, transcriptomic, or multi-omics signature.

Inflammatory and immune markers were the most frequently studied. CRP or hs-CRP, IL-6, LPS, periodontal-pathogen antibodies, MBL, and SIRT1 were evaluated in different clinical settings. The direction and strength of findings were not uniform. For example, Da Venezia et al. did not identify an independent association between periodontal diagnosis and serum hs-CRP-based cardiovascular risk after adjustment, whereas large cohort analyses linked periodontal status or immune-response profiles with later cardiovascular outcomes. These studies differed substantially in population, periodontal definition, endpoint, assay, and confounder control.

Endothelial, vascular, lipid-related, autoimmune, cardiac-stress, and oxidative-stress markers provided additional but non-equivalent evidence. Zhou et al. reported reductions in blood pressure and endothelial microparticles after intensive periodontal therapy. In contrast, Molina et al. observed periodontal improvement without significant between-group differences in FMD or cIMT, and Latorre Uriza et al. reported an imprecise, non-significant adjusted association between periodontitis and cIMT. PCSK9, anti-ApoA-1 IgG, NT-proBNP, and oxidative-stress measures were potentially informative, but each was supported by a limited number of studies and was not externally validated as a periodontitis-specific cardiovascular biomarker.

Only Latorre Uriza et al. and Stănescu et al. directly compared men and women. Their findings concerned selected vascular, inflammatory, periodontal, or oxidative-stress variables and did not demonstrate a validated sex-by-periodontitis biomarker interaction. Da Venezia et al. included women only and cannot support a between-sex inference. No study stratified women by menopausal status. Therefore, the review identifies overlapping candidate-biomarker domains but cannot answer the original question by defining a sex-specific or menopause-specific blood-biomarker signature.

## 4. Discussion

This systematic review synthesized the available evidence on candidate biomarkers linking periodontitis with atherosclerotic and related cardiovascular phenotypes, with particular attention to sex-related findings [64,65,66,67,68,69,70,71,72]. Overall, the included studies suggest that the periodontal–cardiovascular association may involve overlapping inflammatory, immune, endothelial, oxidative, lipid-related, autoimmune, and metabolic pathways. However, the evidence derives predominantly from studies evaluating predefined candidate biomarkers rather than genuine high-throughput proteomic, transcriptomic, metabolomic, or multi-omics platforms [73,74,75,76,77]. The findings should therefore be interpreted as an exploratory mapping of potentially relevant biological pathways rather than as the identification of a validated molecular signature.

The cardiovascular phenotypes investigated were also heterogeneous and included coronary artery disease, myocardial infarction, ischemic stroke, abdominal aortic aneurysm, heart failure, prehypertension, carotid intima-media thickness, and endothelial dysfunction. Although these conditions share inflammatory and vascular mechanisms, they are not clinically interchangeable. Biomarkers recurring across different phenotypes may therefore indicate pathway-level overlap, but they cannot be regarded as disease-specific markers of periodontitis-associated atherosclerosis.

### 4.1. Candidate-Biomarker Evidence and Cross-Phenotype Consistency

Inflammatory and pathogen-related immune markers were the most frequently investigated. Da Venezia et al. assessed serum high-sensitivity C-reactive protein and gingival crevicular fluid CRP in women with different periodontal conditions [53]. Local CRP reflected periodontal inflammatory burden, whereas the study did not establish an independent association between periodontal status and serum hs-CRP after adjustment for relevant variables. Molinsky et al., using the large ARIC cohort, associated periodontal status and edentulism with incident heart failure and evaluated CRP and NT-proBNP [55]. These findings support a relationship between periodontal status, systemic inflammation, and cardiac stress, although heart failure represents a broader cardiovascular phenotype rather than direct evidence of atherosclerotic disease.

Qi et al. investigated circulating IgG antibodies against periodontal microorganisms in relation to cardiovascular and all-cause mortality [56], whereas Salhi et al. evaluated CRP, lipopolysaccharide, and antibodies against *Porphyromonas gingivalis* and *Aggregatibacter actinomycetemcomitans* in patients with abdominal aortic aneurysm [59]. Both studies suggest that pathogen-related immune responses may contribute to systemic vascular inflammation. However, antibody concentrations do not demonstrate active infection or causality, and the post hoc design and selected clinical population of Salhi et al. limit generalizability. Rathnayake et al. identified higher salivary peptidoglycan recognition protein 1 levels in patients with myocardial infarction within the PAROKRANK population [57]. Although the association persisted after adjustment for several covariates, the subgroup design and the use of saliva rather than blood limit its interpretation as a circulating cardiovascular biomarker.

Rughwani et al. evaluated serum and salivary PCSK9 and IL-6 in patients with periodontitis and atherosclerotic cardiovascular disease [58]. Their findings suggest a possible convergence between periodontal inflammation and lipid-regulatory pathways, but the case–control design could not fully separate the contributions of periodontal disease, cardiovascular disease, medication use, and metabolic risk factors. Similarly, Caribé et al. assessed SIRT1 and mannose-binding lectin in patients with and without periodontitis and coronary artery disease [52]. Differences among clinical groups support the involvement of innate immune and metabolic regulation, although the relatively small sample and coexistence of periodontal and coronary conditions prevent attribution of the biomarker changes specifically to periodontitis.

Autoimmune and oxidative mechanisms were addressed by Wick et al. and Stănescu et al. Wick et al. reported a higher prevalence of anti-apolipoprotein A-1 IgG positivity in patients with periodontitis and an association with atherosclerotic burden, particularly among younger participants [61]. This result suggests a possible autoimmune link between periodontal inflammation and lipid-related vascular damage, but its cross-sectional design and subgroup analyses require cautious interpretation. Stănescu et al. evaluated patients with ischemic stroke and identified relationships among periodontal destruction, behavioral risk factors, systemic oxidative imbalance, and sex [60]. The findings support oxidative stress as a shared pathway, although the pilot design and absence of a non-stroke comparison group limit conclusions regarding the independent contribution of periodontitis.

Endothelial and vascular outcomes were examined in one observational study and two periodontal-treatment trials. Latorre Uriza et al. evaluated periodontal status, inflammatory biomarkers, and carotid intima-media thickness [54]. Although some periodontal and inflammatory variables were associated with vascular measurements, the adjusted association between periodontitis and carotid intima-media thickness was not statistically significant, suggesting an important contribution from conventional cardiovascular risk factors. Zhou et al. reported that intensive periodontal therapy in patients with prehypertension improved periodontal parameters and was accompanied by reductions in blood pressure and endothelial microparticles [63]. These findings suggest a potentially modifiable relationship between periodontal inflammation and endothelial injury, but they rely on surrogate vascular outcomes and should be interpreted in light of the trial’s methodological limitations.

Molina et al. evaluated periodontal therapy in patients with severe periodontitis and established cardiovascular disease, assessing flow-mediated dilation, carotid intima-media thickness, and selected serum biomarkers [62]. Periodontal outcomes improved, but significant between-group differences in flow-mediated dilation or carotid intima-media thickness were not demonstrated. This study therefore provides limited evidence of a direct short-term vascular effect and highlights the importance of distinguishing within-group changes from treatment effects supported by between-group comparisons.

Collectively, the 12 studies do not identify a single dominant biomarker or a consistent biomarker panel. Instead, they indicate possible convergence across inflammatory, immune, endothelial, oxidative, autoimmune, lipid-related, and metabolic pathways. The biological plausibility of this overlap is supported by the recurrence of related biomarker domains, but consistency was reduced by differences in populations, cardiovascular phenotypes, periodontal definitions, biological samples, laboratory assays, adjustment strategies, and outcome timing.

### 4.2. Sex-Related Evidence and Menopausal Gap

Sex-related differences in cardiovascular biomarker concentrations provide a biological rationale for investigating whether sex modifies the relationship between periodontitis and cardiovascular disease. However, baseline differences between men and women do not demonstrate a sex-specific periodontal effect. Establishing effect modification requires adequately powered sex-by-periodontitis interaction analyses rather than simple reporting of sex distribution or statistical adjustment for sex.

Only two included studies performed direct male–female comparisons. Latorre Uriza et al. reported sex-related differences in selected inflammatory and vascular variables but did not identify a validated sex-specific periodontal biomarker profile [54]. Stănescu et al. found that male patients with ischemic stroke showed more severe periodontal destruction and a less favorable oxidative-stress profile [60]. These observations are clinically relevant but arose from studies without external validation or formal interaction testing.

Da Venezia et al. included women only and therefore provided female-specific information without permitting comparison between sexes [53]. The remaining studies either reported sex as a demographic characteristic or included it as a covariate but did not provide sufficiently detailed sex-stratified biomarker analyses [52,55,56,57,58,59,61,62,63]. Consequently, the available evidence cannot determine whether the observed biomarker associations differ meaningfully between men and women.

None of the included studies stratified women according to menopausal status. This represents a major research gap because menopausal transition may influence inflammation, lipid metabolism, endothelial function, immune regulation, and periodontal tissue response. The current absence of sex- and menopause-specific evidence should therefore be interpreted as insufficient investigation rather than evidence that biological differences do not exist [78,79,80,81,82,83].

### 4.3. Certainty of Evidence, Limitations, and Research Priorities

The certainty of the evidence is limited by the predominance of observational, cross-sectional, case–control, post hoc, and pilot designs. Residual confounding and participant selection were the most frequent concerns, particularly because age, smoking, diabetes, adiposity, medication use, socioeconomic conditions, oral-health behavior, periodontal severity, and baseline cardiovascular risk were not adjusted consistently [84,85,86,87,88,89]. Objective clinical examinations, laboratory assays, and vascular imaging reduced some concerns regarding outcome measurement, but did not eliminate limitations related to exposure classification, missing data, analytical flexibility, or selection of reported results.

The randomized studies provided more appropriate designs for evaluating the effects of periodontal treatment, but confidence was reduced by incomplete reporting of allocation procedures, possible deviations from intended interventions, multiple endpoints, and reliance on surrogate vascular measures. Findings from studies with greater methodological concerns should therefore be considered exploratory and hypothesis-generating rather than confirmatory [90,91,92,93,94,95,96].

Clinical and methodological heterogeneity further restricted comparability and precluded meaningful quantitative pooling. Periodontitis was defined using different diagnostic criteria, severity thresholds, treatment histories, or pathogen-related measures. Cardiovascular outcomes ranged from established events to subclinical imaging findings and broader cardiovascular conditions [97,98,99,100,101,102]. Biological matrices included serum, blood, saliva, and gingival crevicular fluid, while biomarker assays were not standardized across studies.

A further limitation was the predominance of candidate-biomarker investigations. Such studies provide focused mechanistic information but cannot capture the broader molecular networks detectable through high-throughput proteomics, transcriptomics, metabolomics, or integrated multi-omics [103,104,105,106,107]. The broad eligibility framework of this review was necessary because directly relevant studies were scarce, but it also increased heterogeneity and required the inclusion of some cardiovascular phenotypes that provide indirect rather than strictly atherosclerosis-specific evidence [108,109,110,111,112].

Future studies should use prospective, adequately powered cohorts with standardized periodontal and cardiovascular phenotyping [113,114,115,116,117]. Comprehensive blood-based molecular profiling should be integrated with vascular imaging and prespecified adjustment for major cardiovascular, metabolic, behavioral, pharmacological, and socioeconomic confounders. Analyses should formally test sex-by-periodontitis interactions and report results separately for men, premenopausal women, and postmenopausal women [118,119,120,121,122]. Interventional trials should prioritize between-group effects, standardized follow-up, blinded outcome assessment where feasible, and clinically meaningful vascular outcomes in addition to surrogate biomarkers [123,124,125,126].

In conclusion, the available literature supports the biological plausibility of a periodontal–cardiovascular relationship involving several interconnected biomarker domains. Nevertheless, heterogeneity, risk of bias, limited use of high-throughput approaches, and inadequate sex- and menopause-specific analyses prevent the identification of a causal or clinically validated biomarker signature [127,128,129,130,131,132,133,134,135]. The current evidence is therefore most useful for identifying overlapping biological pathways and defining priorities for future validation-oriented research.

## 5. Conclusions

This systematic review identifies overlapping candidate-biomarker pathways at the intersection of periodontitis and atherosclerotic or related cardiovascular phenotypes. The most frequently represented domains were inflammation and immunity, endothelial or vascular injury, oxidative stress, lipid and autoimmune regulation, and cardiac stress. These findings support biological plausibility, but they do not establish that the reported biomarkers are specific mediators of the periodontal–cardiovascular association or that they improve clinical prediction.

The evidence base consisted mainly of observational studies measuring single analytes or small predefined panels, with substantial heterogeneity in periodontal definitions, cardiovascular outcomes, biological matrices, assays, and confounder adjustment. Only two studies directly compared men and women; neither demonstrated a validated sex-by-periodontitis biomarker interaction, and no study stratified women by menopausal status. A sex-specific or menopause-specific biomarker signature therefore cannot be defined from the current literature.

Future research should combine standardized periodontal assessment, cardiovascular imaging, comprehensive blood-based omics, prespecified sex-interaction analyses, direct collection of menopausal and reproductive variables, correction for multiple testing, and independent validation. Until such evidence is available, the biomarkers summarized here should be regarded as hypothesis-generating rather than clinically actionable.

## Figures and Tables

**Table 1:** Database-specific search strategies.

Database	Search Field	Search Strategy
PubMed	Title/Abstract/MeSH-compatible terms	(“periodontitis” OR “periodontal disease” OR “periodontal inflammation” OR “periodontal infection” OR “periodontal pathogens” OR “*Porphyromonas gingivalis*” OR “*Aggregatibacter actinomycetemcomitans*” OR “clinical attachment loss” OR “bleeding on probing”) AND (“atherosclerosis” OR “atherosclerotic plaque” OR “cardiovascular disease” OR “coronary artery disease” OR “myocardial infarction” OR “stroke” OR “ischemic stroke” OR “carotid intima-media thickness” OR “carotid plaque” OR “endothelial dysfunction” OR “hypertension” OR “prehypertension” OR “vascular inflammation”) AND (“biomarker” OR “biomarkers” OR “blood biomarker” OR “serum biomarker” OR “plasma biomarker” OR “inflammatory marker” OR “immune marker” OR “endothelial marker” OR “omics” OR “proteomic” OR “proteomics” OR “proteome” OR “immunoproteomic” OR “immunoproteomics” OR “transcriptomic” OR “transcriptomics” OR “transcriptome” OR “gene expression” OR “RNA sequencing” OR “multi-omics” OR “targeted proteomics”)
Scopus	TITLE-ABS-KEY	TITLE-ABS-KEY (periodontitis OR “periodontal disease” OR “periodontal inflammation” OR “periodontal infection” OR “periodontal pathogens” OR “*Porphyromonas gingivalis*” OR “*Aggregatibacter actinomycetemcomitans*” OR “clinical attachment loss” OR “bleeding on probing”) AND TITLE-ABS-KEY (atherosclerosis OR “atherosclerotic plaque” OR “cardiovascular disease” OR “coronary artery disease” OR “myocardial infarction” OR stroke OR “ischemic stroke” OR “carotid intima-media thickness” OR “carotid plaque” OR “endothelial dysfunction” OR hypertension OR prehypertension OR “vascular inflammation”) AND TITLE-ABS-KEY (biomarker OR biomarkers OR “blood biomarker” OR “serum biomarker” OR “plasma biomarker” OR “inflammatory marker” OR “immune marker” OR “endothelial marker” OR omics OR proteomic OR proteomics OR proteome OR immunoproteomic OR immunoproteomics OR transcriptomic OR transcriptomics OR transcriptome OR “gene expression” OR “RNA sequencing” OR “multi-omics” OR “targeted proteomics”)
Web of Science	Topic field (TS)	TS = (periodontitis OR “periodontal disease” OR “periodontal inflammation” OR “periodontal infection” OR “periodontal pathogens” OR “*Porphyromonas gingivalis*” OR “*Aggregatibacter actinomycetemcomitans*” OR “clinical attachment loss” OR “bleeding on probing”) AND TS = (atherosclerosis OR “atherosclerotic plaque” OR “cardiovascular disease” OR “coronary artery disease” OR “myocardial infarction” OR stroke OR “ischemic stroke” OR “carotid intima-media thickness” OR “carotid plaque” OR “endothelial dysfunction” OR hypertension OR prehypertension OR “vascular inflammation”) AND TS = (biomarker OR biomarkers OR “blood biomarker” OR “serum biomarker” OR “plasma biomarker” OR “inflammatory marker” OR “immune marker” OR “endothelial marker” OR omics OR proteomic OR proteomics OR proteome OR immunoproteomic OR immunoproteomics OR transcriptomic OR transcriptomics OR transcriptome OR “gene expression” OR “RNA sequencing” OR “multi-omics” OR “targeted proteomics”).

**Table 2:** Risk-of-bias assessment of the included studies according to study design. Panel A: observational studies assessed using ROBINS-E; Panel B: randomized trials assessed using RoB 2.

**Panel A—ROBINS-E**								
**Authors**	**D1**	**D2**	**D3**	**D4**	**D5**	**D6**	**D7**	**Overall**
Caribé et al. (2020) [52]	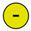	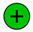	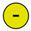	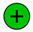	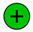	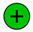	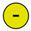	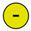
Da Venezia et al. (2021) [53]	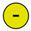	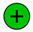	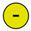	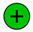	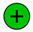	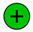	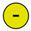	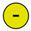
Latorre Uriza et al. (2024) [54]	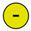	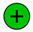	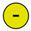	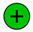	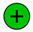	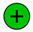	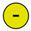	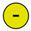
Molinsky et al. (2022) [55]	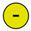	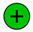	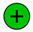	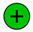	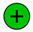	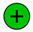	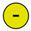	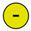
Qi et al. (2020) [56]	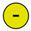	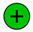	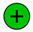	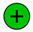	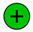	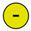	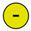	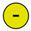
Rathnayake et al. (2022) [57]	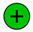	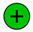	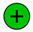	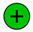	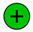	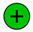	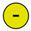	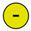
Rughwani et al. (2022) [58]	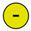	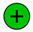	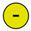	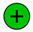	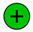	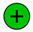	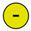	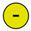
Salhi et al. (2022) [59]	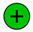	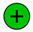	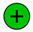	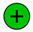	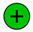	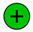	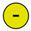	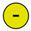
Stănescu et al. (2020) [60]	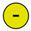	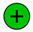	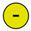	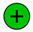	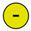	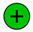	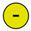	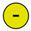
Wick et al. (2013) [61]	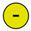	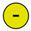	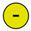	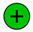	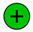	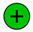	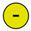	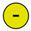
**Panel B—RoB 2**								
**Authors**	**D1**	**D2**	**D3**	**D4**	**D5**	**Overall**		
Molina et al. (2025) [62]	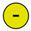	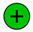	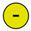	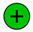	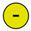	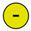		
Zhou et al. (2017) [63]	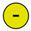	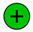	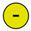	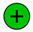	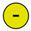	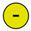		
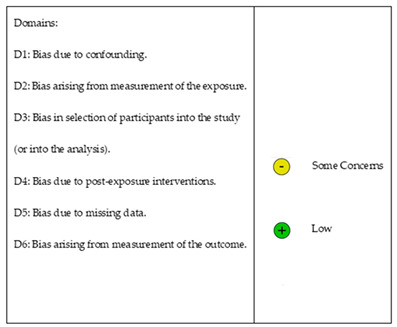								

**Table 3:** Study characteristics, biomarker assessment and sex-specific information of included studies.

Study	Study Design and Sample	Cardiovascular Phenotype	Periodontal Assessment/ Exposure	Biological Matrix and Biomarker Assessment	Main Findings and Interpretive Caveats	Sex and Menopause-Related Information
Caribé et al. (2020) [52]	Prospective case–control study; 78 patients with and without periodontitis and coronary artery disease	Coronary artery disease	Clinical periodontal assessment and nonsurgical periodontal treatment	Serum SIRT1 and MBL	Periodontal treatment was associated with lower MBL and CRP levels and higher SIRT1 levels. The non-randomized design and selected clinical sample limit causal interpretation.	Sex distribution reported; no sex-stratified biomarker estimates; menopausal status not reported
Da Venezia et al. (2021) [53]	Cross-sectional study; 112 adult women with periodontal conditions	Cardiovascular risk in women	Periodontal diagnosis and local inflammatory burden	Serum and gingival crevicular fluid; hs-CRP and gingival crevicular fluid CRP	Periodontal diagnosis was not independently associated with serum hs-CRP-based cardiovascular risk after adjustment; BMI was the principal determinant of the serum risk category. Gingival crevicular fluid CRP mainly reflected local inflammation.	Women-only study; no male comparator and no stratification according to menopausal status
Latorre Uriza et al. (2024) [54]	Cross-sectional study; 75 participants	Subclinical atherosclerosis assessed using carotid intima-media thickness	Clinical periodontal status	Blood-based inflammatory biomarkers and Carotid intima-media thickness	The adjusted association between periodontitis and cIMT (carotid intima-media thickness) was not statistically significant and was imprecise. Men had higher left cIMT and higher IL-8 and TNF-α levels, but a sex-by-periodontitis interaction was not tested.	Direct male–female comparisons reported; no periodontitis-specific interaction analysis; menopausal status not reported.
Molina et al. (2025) [62]	Randomized pilot clinical trial; 35 patients with stage III/IV periodontitis and established cardiovascular disease	Established cardiovascular disease and vascular dysfunction	Periodontal therapy	Serum; FMD, cIMT and selected inflammatory and vascular biomarkers	Periodontal outcomes improved after therapy, but no significant between-group differences were observed for FMD or cIMT. The pilot study was underpowered for vascular endpoints.	Sex distribution reported; no sex-stratified treatment effect; menopausal status not reported.
Molinsky et al. (2022) [55]	Prospective cohort study; 6.707 participants from the ARIC cohort	Incident heart failure and cardiovascular risk	Periodontal status and edentulism	Blood; CRP and NT-proBNP	Poor periodontal status was associated with incident heart failure and unfavorable CRP and NT-proBNP trajectories. Heart failure represents a broader cardiovascular phenotype rather than a direct measure of atherosclerosis.	Sex included in adjusted models; no sex-stratified periodontal biomarker signature; menopausal status not reported.
Qi et al. (2020) [56]	Prospective cohort study; 6.491 participants	Cardiovascular and all-cause mortality	Serum immune response profiles against periodontal microorganisms	Serum; IgG antibodies against periodontal microorganisms	Periodontal IgG antibody clusters were associated with mortality outcomes in an observational analysis. Antibody levels represent exposure or immune-response proxies rather than discovery-scale omics measurements.	Sex included in adjustment; no sex-stratified antibody–outcome estimates; menopausal status not reported.
Rathnayake et al. (2022) [57]	Case–control subgroup analysis; 400 participants from the PAROKRANK population	Myocardial infarction	Periodontal inflammation and periodontal status	Saliva; PGLYRP1	Salivary PGLYRP1 was higher in patients with myocardial infarction even after adjustment. This represents oral-fluid rather than blood-based evidence and derives from a subgroup case–control analysis.	Adjusted for sex; no sex-stratified PGLYRP1 estimates; menopausal status not reported
Rughwani et al. (2022) [58]	Case–control study; 76 participants	Atherosclerotic cardiovascular disease	Periodontal inflammation	Serum and saliva; PCSK9 and IL-6	Serum and salivary PCSK9 and IL-6 differed across periodontal and cardiovascular disease groups. The small case–control sample and multiple comparisons limit reproducibility and causal interpretation.	Sex distribution reported; no sex-stratified biomarker estimates; menopausal status not reported.
Salhi et al. (2022) [59]	Post hoc cross-sectional study; 61 patients with abdominal aortic aneurysm	Abdominal aortic aneurysm	Periodontitis-related inflammatory and microbial burden	Blood and oral samples; CRP, LPS and antibodies against *P. gingivalis* and *A. actinomycetemcomitans*	CRP, LPS, and periodontal-pathogen antibodies were evaluated in a selected vascular population. The post hoc design provides indirect, non-causal evidence with limited generalizability.	Sex distribution reported; no sex-stratified biomarker analysis; menopausal status not reported
Stănescu et al. (2020) [60]	Pilot cohort study; 93 patients with ischemic stroke	Ischemic stroke	Periodontal destruction parameters	Blood; systemic oxidative- stress markers	Men with ischemic stroke had more severe periodontal destruction and a less favorable oxidative-stress profile. Behavioral risk factors also differed by sex and may have confounded the comparison.	Direct male–female comparison reported; no formal sex-by-periodontitis interaction and no menopausal data.
Wick et al. (2013) [61]	Cross-sectional case–control study; 176 participants	Atherosclerotic burden	Periodontitis status	Serum; anti-ApoA-1 IgG	Anti-ApoA-1 IgG was more frequent in patients with periodontitis and was associated with atherosclerotic burden, particularly in a younger subgroup. The cross-sectional design and subgroup analyses limit inference.	Sex distribution reported; no sex-stratified biomarker analysis; menopausal status not reported.
Zhou et al. (2017) [63]	Randomized controlled trial; 95 prehypertensive patients with periodontitis	Prehypertension/endothelial dysfunction	Intensive periodontal therapy	Blood-based endothelial assessment and endothelial microparticles	Intensive periodontal therapy reduced blood pressure and endothelial microparticles compared with control treatment. The vascular outcomes were surrogate endpoints, and sex-specific treatment effects were not evaluated	Sex distribution reported; no sex-stratified treatment effect; menopausal status not reported

## Data Availability

No new data were created or analyzed in this study. Data sharing is not applicable to this article.

## Abbreviations

The following abbreviations are used in this manuscript:

anti-ApoA-1 IgG	anti-Apolipoprotein A-1 Immunoglobulin G
ARIC	Atherosclerosis Risk in Communities
BMI	Body Mass Index
cIMT	Carotid intima-media thickness
CRP	C-reactive protein
FMD	Flow Mediated Dilation
hs-CRP	High-sensitivity C-reactive protein
IgG	Immunoglobulin G
IL-6	Interleukin-6
IL-8	Interleukin-8
LPS	Lipopolysaccharide
MBL	Mannose-binding lectin
NT-proBNP	N-terminal pro-B-type natriuretic peptide
PAROKRANK	Periodontitis and Its Relation to Coronary Artery Disease
PCSK9	Proprotein convertase subtilisin/kexin type 9
PGLYRP1	Peptidoglycan recognition protein 1
SIRT1	sirtuin 1
